# Roles of mechanosensitive channel Piezo1/2 proteins in skeleton and other tissues

**DOI:** 10.1038/s41413-021-00168-8

**Published:** 2021-10-20

**Authors:** Lei Qin, Tailin He, Sheng Chen, Dazhi Yang, Weihong Yi, Huiling Cao, Guozhi Xiao

**Affiliations:** 1 Department of Orthopedics, Huazhong University of Science and Technology Union Shenzhen Hospital, Shenzhen, Guangdong China; 2 Department of Biochemistry, School of Medicine, Guangdong Provincial Key Laboratory of Cell Microenvironment and Disease Research, Shenzhen Key Laboratory of Cell Microenvironment, Southern University of Science and Technology, Shenzhen, Guangdong China; 3 Department of Orthopedics, Union Hospital, Tongji Medical College, Huazhong University of Science and Technology, Wuhan, Hubei China

**Keywords:** Bone quality and biomechanics, Bone

## Abstract

Mechanotransduction is a fundamental ability that allows living organisms to receive and respond to physical signals from both the external and internal environments. The mechanotransduction process requires a range of special proteins termed mechanotransducers to convert mechanical forces into biochemical signals in cells. The Piezo proteins are mechanically activated nonselective cation channels and the largest plasma membrane ion channels reported thus far. The regulation of two family members, Piezo1 and Piezo2, has been reported to have essential functions in mechanosensation and transduction in different organs and tissues. Recently, the predominant contributions of the Piezo family were reported to occur in the skeletal system, especially in bone development and mechano-stimulated bone homeostasis. Here we review current studies focused on the tissue-specific functions of Piezo1 and Piezo2 in various backgrounds with special highlights on their importance in regulating skeletal cell mechanotransduction. In this review, we emphasize the diverse functions of Piezo1 and Piezo2 and related signaling pathways in osteoblast lineage cells and chondrocytes. We also summarize our current understanding of Piezo channel structures and the key findings about *PIEZO* gene mutations in human diseases.

## Introduction

Mechanotransduction is a fundamental ability that allows living organisms to receive and respond to physical signals from the internal and external environment and has been found and characterized in all five kingdoms of life. For bacteria or other simple organisms, mechanotransduction is required to sense stretching, osmotic pressure, and other mechanical forces.^1^ In mammals, mechanotransduction is involved in many physiological processes,^2,3^ such as touch, gravity, proprioception, sound, air flow, vascular development, and blood pressure. Successful and precise mechanotransduction is essential for proper organ function, whereas abnormal or faulty mechanotransduction could lead to a wide array of diseases, such as deafness,^4^ cardiovascular diseases,^5^ metabolic defects,^3^ fibrosis,^2^ cancer metastasis,^6^ neuronal disorders,^7^ and osteoporosis.^8^ The mechanotransduction process requires a range of special proteins termed mechanotransducers to convert mechanical forces into biochemical signals, which further induce a series of sequential reactions in cells. As the primary mechanism for mechanotransduction, mechanically activated (MA) ion channels can be directly stimulated by mechanical forces that are applied to cell envelopes with fast and efficient responses of either cell membrane excitation or the activation of intracellular signaling.^1,9^ Before 2010, our understanding of the gating mechanism of MA channels was mostly from bacterial work. The discovery of the Piezo channel family opens a new area in the field to study the components and functions of eukaryotic MA channels.

In 2010, a tour de force of work by Patapoutian and colleagues uncovered a new group of mammalian MA channels named the Piezo family.^10^ This groundbreaking study attracted great attention from a number of researchers worldwide to study the functions of the two family members Piezo1 and Piezo2 in different tissues and organs. During the past 10 years, studies using experimental mouse models have indicated that Piezo1 is mainly expressed in nonexcitable cell types^11^ and is critical for transducing mechanical forces applied externally and internally at the plasma membrane.^12^ In contrast, Piezo2 is primarily expressed in sensory neurons,^11^ including somatosensory ganglia, outer hair cells, enterochromaffin cells of the gut, and Merkel cells, but not somatic cells.^13^ Cumulative evidence suggests that Piezo1 is required for vascular development and function,^14^ red blood cell volume regulation,^15^ epithelial homeostasis,^16^ the lineage choice of neural stem cells,^17^ axon growth,^18^ and urinary osmolarity.^19^ Piezo2 has essential roles in sensory processes, such as gentle touch sensation,^20^ mechanical nociception^21^ and proprioception,^22^ and indispensable functions in auditory sensation,^23^ gastrointestinal physiology,^24,25^ and respiratory physiology.^26^ More recently, the predominant contributions of the Piezo family were reported to occur in the skeletal system, especially in bone development and mechano-stimulated bone homeostasis.^27–30^ These studies demonstrate the important functions of Piezo proteins in regulating stem cell fate and osteoblast lineage cell mechanotransduction. Here we summarize our current understanding of Piezo family functions in different tissue backgrounds with a special highlight of their roles in bone cells and chondrocytes. We also briefly review the key findings of PIEZO channels in human diseases.

## Discovery of Piezo proteins

To search for new MA ion channels, in 2010, the research team of Patapoutian established a simple but efficient cell culture platform to test the MA current by pressing cultured cells with a glass probe that simultaneously records the whole-cell configuration through a patch clamp.^10^ By using of this platform, they found that a mouse neuroblastoma cell line, Neuro2A cells, displayed the most consistent MA currents with relatively fast adaptation in response to stimuli. Microarray and small interfering RNA (siRNA) screening with Neuro2A cells found Fam38A (family with sequence similarity 38) to be indispensable for MA currents. Previously, Fam38A (also called KIAA0233 or Mib) was reported to be associated with astrocyte activation in senile plaque-associated Alzheimer’s disease progression^31^ and integrin activation in epithelial cells.^32^ For the first time, the Fam38A gene was reported to have an essential function in pressure-induced MA currents. Based on these facts, the Patapoutian group named this gene *Piezo1*, from the Greek “pίesh” (píesi), meaning pressure. *Piezo2* was later identified as a gene homologous to *Piezo1* in sequence homology analysis.^10^

Piezo proteins are large transmembrane (TM) proteins that are evolutionarily conserved among various species, including animals, plants, and protozoa, but not yeast and bacteria.^10,33^ Among invertebrates, *Drosophila melanogaster* has only one Piezo member (DmPiezo, known as CG8486).^21^ The amino acid (aa) sequence of DmPiezo is 24% identical to that of mammalian Piezo proteins and is able to induce MA currents in cells.^34^ DmPiezo knockout (KO) larvae showed reduced behavioral responses (rollover behavior) to noxious mechanical stimuli (poking) but no difference in responses to another noxious stimulus or touch.^21^ The responsive organ in *D. melanogaster* could be the sensory neurons in the larval body wall.^21,35^ In vertebrates, zebrafish have three Piezo members, i.e., Piezo1, Piezo2A, and Piezo2B. The aa sequence of the zebrafish Piezo1 protein is 59.2% identical to that of the human Piezo1 protein, and Piezo2A and Piezo2B share 66.9% and 62.6% of their aa sequences with the human Piezo2 protein, respectively.^36^ It was reported that Piezo2B expression in zebrafish embryonic Rohon–Beard cells was essential for mediating light touch responses.^36^

In mammals, there are two Piezo proteins, Piezo1 and Piezo2. The human *PIEZO1* gene, also known as *DHS/FAM38A/LMPH3/LMPHM6*, is located in region 16q24.3 of chromosome 16. It contains 51 exons that encode the PIEZO1 protein, composed of 2 520 aa residues (NCBI database). Human *PIEZO1* is a highly polymorphic gene that has a number of variants.^37^ The human *PIEZO2* gene, also known as *C18orf30/C18orf58/DA3/DA5/DAIPT/FAM38B/FAM38B2/HsT748/HsT771/MWKS*, is located in region 18p11.22-p11.21 on chromosome 18. It contains 57 exons that encode the PIEZO2 protein, composed of 2 752 aa residues (NCBI database). Human genomic studies have demonstrated that both loss- and gain-of-function human *PIEZO genes* cause various defects in humans,^38–40^ which emphasizes the broad involvement and essential roles of PIEZO in the development and homeostasis of the human body and will be discussed in a later section of this review. The mouse *Piezo1* gene, also known as *9630020g22/Fam3/Fam38a/Pie/mKIAA0233*, is located in region 8 E1 on chromosome 8. The mouse *Piezo1* gene contains 53 exons that encode the Piezo1 protein, which is composed of 2 547 aa residues (NCBI database). The mouse *Piezo2* gene, also known as *5930434P17/9030411M15Rik/9430028L06Rik/Fam38/Fam38b/Fam38b2/Pie*, is located in region 18 E1 of chromosome 18, which contains 56 exons encoding the Piezo2 protein, which consists of 2 822 aa (NCBI database). The breakthrough discovery of mammalian Piezo proteins has opened a new era of mechanical transduction research. Enormous efforts have been made to illustrate their physiological and pathological significance based on the properties of MA ion channels.

## Molecular structures of Piezo proteins

In parallel with the considerable studies focused on the biological functions of Piezo1 and Piezo2 as mechanotransducers,^10,14,21,41^ great attention has also been given to deciphering their molecular composition and structures. Coste et al. first reported that both Piezo1 and Piezo2 are unusually large proteins with predicted lengths of 2 100–4 700 aa and contain 24–36 TM domains,^10^ which makes Piezo the largest plasma membrane ion channel complex identified thus far.^34^ Piezo proteins have unique sequences without any repetitive sequence patterns.^33^ In addition to their giant molecular weight, Piezo proteins lack apparent sequence homology with any other known ion channels or proteins.^33,34^

**Fig. 1:**
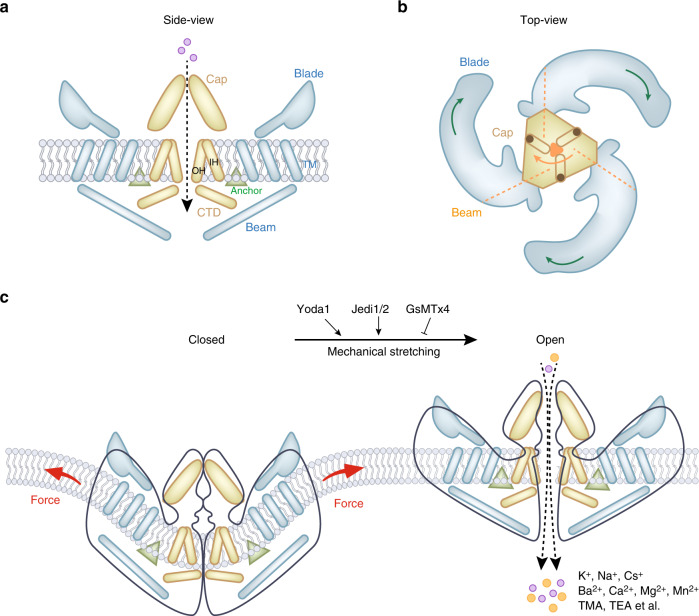
Mouse Piezo1 protein has a three-bladed, propeller-shaped homotrimeric architecture. **a** Side view of mouse Piezo1 channel. Piezo1 consists of a central ion-conducting pore modulus (yellow components) and the peripheral mechanotransduction modulus (blue component). The pore module contains the extracellular Cap structure, the transmembrane pore formed from three pairs of TMs, and the intracellular C-terminal domain (CTD). The peripheral mechanotransduction modulus includes a long beam-like structure, a peripheral blade, and a unique anchor domain. The anchor domain formed from a hairpin structure is connected to the CTD plane by the inner helix (IH) and outer helix (OH) pair, which maintains the integrity of the channel. The long beam structure supports and bridges the blade into the central pore module. **b** Top view of mouse Piezo1 channel. The large extracellular blade domains can curve the plasma membrane, and the three blades are assembled into functional trimers. **c** Mammalian Piezo1 proteins can be directly gated by membrane stretching, which is conserved throughout evolution. Yoda1 and Jedi1/2 are chemical activators of Piezo channels, and GsMTx4 is an antagonist of the Piezo1 channel. Piezo channels are nonselective cationic mechanosensitive channels that are permeable to alkali ions (K^+^, Na^+^, and Cs^+^), divalent cations (Ba^2+^, Ca^2+^, Mg^2+^, and Mn^2+^), and several organic cations (tetramethyl ammonium (TMA), tetraethyl ammonium (TEA)).^56^ Illustrations were modified from Wang et al.^52^ and Jiang et al.^11^

The molecular structure of the full-length (2 547 aa) mouse Piezo1 channel was first revealed by cryo-electron microscopy (cryo-EM) at a resolution of 4.8 Å in 2015.^42^ Later, the mouse Piezo1 structure was solved at higher resolution (3.8 Å^43^ and 3.97 Å^44^) by cryo-EM. Structurally, high-resolution cryo-EM techniques have resolved the “nanobowl” configuration of Piezo proteins,^11^ which deform lipid bilayers locally into a dome shape^45^ (Fig. 1). Overall, the mouse Piezo1 protein has a three-bladed, propeller-shaped homotrimeric architecture (Fig. 1a, b),^42,44^ composing a unique 38-TM-helix topology^44^ in each subunit with a total of 114 TM helices in the trimeric channel complex.^44^ For each subunit, Piezo1 is composed of a central ion-conducting pore modulus and three peripheral mechanotransduction moduli (Fig. 1a).^44,46^ These distinct and separable moduli of Piezo1 are responsible for different channel functions.^47^ The pore module contains the extracellular Cap structure, the TM pore formed from three pairs of TMs, and the intracellular C-terminal domain (CTD).^48^ Functional studies of the Piezo1 modulus found that residues 2 189–2 547 of mouse Piezo1 inside the pore module govern fundamental pore properties, including unitary conductance, ion selectivity, and pore blockage.^47^ A minimal region of the Piezo1 protein (aa 2 172–2 547) was reported to be able to fold and function as a channel pore domain.^49^ The peripheral mechanotransduction modulus includes a long beam-like structure, a peripheral blade, and a unique anchor domain.^44,48^ Importantly, the long beam structure supports and bridges the blade into the central pore module.^48^ The large extracellular blade domains can curve the plasma membrane (Fig. 1c), and the three blades are assembled into functional trimers.^46^ Furthermore, the anchor domain formed from a hairpin structure is connected to the CTD plane by the inner helix and outer helix pair, which maintains the integrity of the channel.^44^ Functional studies showed that residues 1–2 190 of the mechanotransduction modulus confer mechanosensitivity to trimeric channel pores.^47^ The structure of the mechanotransduction modulus is essential for Piezo1 mechanical activation.^44^

The structure of Piezo2 is rather similar to that of Piezo1, sharing approximately 42% sequence homology.^10^ The Piezo2 channel is also a three-bladed, propeller-like trimer that comprises 114 TM helices (38 per protomer).^50^ Compared to Piezo1, there are several charged residues at the interface between the beam and the CTD in the Piezo2 channel, which maintain the mechanosensitivity.^50,51^ In addition, extra constriction sites between the two inner helices of the Piezo2 channel were reported, which resulted in the Piezo2 channel having a narrower cavity than the Piezo1 channel.^50–52^ These structural differences between Piezo1 and Piezo2 suggest that the TM site might serve as a TM gate that controls the channel permeability and mechanosensitivity.^50^ Detailed structural illustrations and discussion of Piezo proteins are presented in three recently published review articles.^11,46,53^

The structures of proteins determine their functions. The structural data of the Piezo1 channel reveal a particular lever-like topological feature for mechanosensitivity (Fig. 1c).^44^ The extracellular blade structures are coupled with the central “cap” domain through the beams (residues H1300–S1362) to form lever-like apparatuses.^54,55^ Moreover, three sets of lever-like apparatuses constitute enormous three-bladed, propeller-like machinery to serve as mechanotransducers.^45,48^ These lever-like apparatuses enable Piezo channels with effective conformational changes from blades to a relatively slight opening of the central pore that allows cation-selective permeation (Fig. 1c).^44^ Piezo channels are nonselective cationic mechanosensitive channels. Human Piezo1 channels are permeable for monovalent ions (such as alkali ions, K^+^, Na^+^, and Cs^+^), divalent ions (Ba^2+^, Ca^2+^, Mg^2+^, and Mn^2+^), and several organic cations (tetramethyl ammonium, tetraethyl ammonium).^56^

Piezo channels show rapid activation and voltage-dependent inactivation, and their kinetics are largely consistent across diverse cell types.^57^ The activation and regulation of Piezo channels are regulated by both solo mechanical stimulation and protein–protein interactions. On the one hand, mammalian Piezo1 proteins can be directly gated by membrane stretch, a feature that is conserved throughout evolution.^42,47^ Piezo proteins can form discrete channels in the absence of other pour-binding auxiliary proteins or cytoskeletal elements for channel activation.^34,58^ In cultured HEK293 cells, which are largely free of cytoskeleton, the cell membrane blebs induced by lipid bilayer tension can activate Piezo1.^59^ In a droplet lipid bilayer system, Piezo1 was activated by an osmotic gradient without any further intracellular components.^60^ Furthermore, Piezo channels have been reported to be stimulated by various mechanical cues,^61^ including membrane stretching,^10^ cell indentation,^10^ fluid shear flow,^62,63^ osmotic stress,^60^ substrate stiffness,^17^ matrix roughness,^64^ and environmental confinement.^65^

The activation of Piezo channels has been reported to be regulated by several associated proteins. Mouse Piezo1 has a direct protein–protein interaction with the endoplasmic reticulum (ER) Ca^2+^ pump sarco/ER Ca^2+^ ATPase (SERCA), which can regulate Piezo1 mechanogation (i.e., the ability of channel opening upon mechanical stimulation) through its binding site at the intracellular linker region of Piezo1.^66^ Polycystin 2 was reported to interact with Piezo proteins and inhibit the activation of Piezo channels.^67^ Stomatin-like protein 3 can interact with Piezo proteins to sensitize cells to mechanical stimulation.^68^ Moreover, the C-terminal part of Piezo1 could interact with trefoil factor family 1 to participate in metastasis.^69^ Recently, Wang et al. reported direct interactions between E-cadherin and Piezo1 channels at the cap domain and intracellular gating components.^70^ This finding uncovers long-distance mechanogation of Piezo channels within a single cell or between two adjacent cells through E-cadherin-mediated adherent junctions (AJs).^70^ With these interactions, Piezo proteins also participate in multiple cellular dynamics, such as cell proliferation, elongation, and migration.^71^

Although Piezo channels are mainly activated by mechanical stimuli, there are several small peptides that can regulate the function of Piezo channels. A large-scale compound screening study identified a small synthetic molecule termed Yoda1 (2-[5-[[(2,6-dichlorophenyl)methyl]thio]-1,3,4-thiadiazol-2-yl]-pyrazine) as the first chemical activator for both human and mouse Piezo1.^72^ Mechanistically, Yoda1 serves as an agonist and affects the sensitivity and inactivation kinetics of the mechanically induced responses of Piezo1 channels.^72^ Other Piezo1-specific pharmacological activators that were identified by high-throughput screening include Jedi1 and Jedi2.^55^ Jedi1/2 share no structural similarity to Yoda1 but activate both human and mouse Piezo1 in a dose-dependent manner.^55^ Functional data suggest that these two Piezo1 activators modulate Piezo1 channels by different mechanisms: Jedi1/2 can activate Piezo1 by acting on the upstream blade, whereas Yoda1 acts on the downstream beam in modulating Piezo1 channel activities.^55^ A spider venom peptide named GsMTx4, which selectively inhibits cationic MA channels,^73^ is an antagonist that inhibits both Piezo1^74^ and Piezo2^75^ channels. The mechanism could be related to GsMTx4 penetration upon membrane expansion, which leads to partial relaxation of the outer monolayer of the plasma membrane and reduces the effective magnitude of the stimulus acting on Piezo channel mechanogating.^73,76^

## Piezo proteins in the bone

In vertebrates, bone is a highly specialized and complex tissue that is well constructed to protect internal organs, support muscles, and enable structure and locomotion.^77^ Bone tissue develops from mesenchymal stem cells (MSCs), which can differentiate into chondrocytes or osteoblast lineage cells depending on the cell fates and sites of the skeleton.^78^ Based on their differentiation sequence, osteoblast lineage cells include osteoblast progenitor cells, osteoblasts, and osteocytes. Bone is a highly dynamic connective tissue. During constant bone remodeling, bone mass maintenance is tightly associated with the removal of old and damaged bone by bone-resorbing osteoclasts, which are derived from bone marrow monocytes (BMMs), and the formation of new bone by osteoblasts, which are differentiated from MSCs. To achieve bone homeostasis, the balance between bone formation and bone resorption is largely orchestrated by osteocytes embedded in the bone matrix.^79–81^

It has been widely accepted that bone actively responds to mechanical load. More than 100 years ago, a masterpiece stating the relationship between bone growth and mechanical load was published by the German surgeon Julius Wolff. This golden rule in bone mechanobiology is “Wolff’s law”, which states that bone grows and remodels in response to applied forces.^81,82^ Evidence has shown that major skeletal cells, including MSCs,^83^ osteoblast lineage cells,^84^ and chondrocytes,^85^ are mechanosensitive cells. Mechanical stimulus has been widely recognized as a vital element for embryonic bone formation,^86^ postnatal bone development,^87,88^ and adult bone maintenance^89,90^ and repair.^91^ Recent advances in skeletal genetics and molecular biology have demonstrated that Piezo proteins are greatly involved in bone development and mechanical responses.

In the bone tissue, the expression of both Piezo1 and Piezo2 was detected, but *Piezo1* mRNA had higher expression than Piezo2 in osteoblasts and osteocytes.^92^ During embryonic bone development, whole-mount in situ hybridization of mouse limb buds showed that both *Piezo1* and *Piezo2* were expressed in the forming buds from embryonic day E12.5 to E14.5 with different distribution patterns. *Piezo1* was mainly expressed in the interdigit region, whereas *Piezo2* was primarily expressed in the forming digit and wrist.^29^ Furthermore, fluorescent reporters showed that Piezo1 protein was detected in the connecting tissue, associated muscles, and differentiating Osterix-positive osteoblasts at the primary ossification centers of E13.5 and E15.5 embryos and postnatal P0 pups during long bone development.^29^ Comparably, Piezo2 protein was found to be expressed in multiple cell types, such as Osterix-positive osteoblasts, growth plate chondrocytes, tendons, connective tissues in the muscle, and skin cells.^29^ During postnatal development, Piezo1 expression was progressively upregulated in young mice.^29^ However, the expression of *Piezo2* mRNA in cortical bone was reduced as the mice grew.^29^ Consistently, the expression level of Piezo1 was much higher than that of Piezo2 in cortical samples from young adult mice.^27^ Moreover, in vitro cell differentiation studies conducted with primary MSCs and BMMs revealed that Piezo1 was highly expressed in early differentiating osteoblast progenitors, whereas expression of Piezo2 was induced during osteoclastogenic differentiation.^93^ These observations suggest that Piezo1 and Piezo2 may play distinct roles in the regulation of bone remodeling.

Because global deletion of Piezo1 leads to embryonic lethality in mice,^94^ studies focused on the functions of Piezo1 in skeletal tissues utilize tissue-specific deletion at different bone developmental stages (Table 1). Two research groups showed that deletion of Piezo1 in limb and head MSCs using *Prx1-Cre* in mice (*Piezo1*^*Prx1*^) resulted in severe bone developmental defects.^29,30^ Even though there was no marked skeletal difference between *Piezo1*^*Prx1*^ mice and their control littermates at E17.5,^30^ newborn (P0) *Piezo1*^*Prx1*^ mice displayed reduced bone formation and multiple bone fractures in the forelimbs (radius and ulna).^29^ Interestingly, the hindlimbs (femur and tibia), which have higher bone mass than the forelimbs, showed only a subtle skeletal phenotype at P0.^30^ However, at P3, *Piezo1*^*Prx1*^ pups displayed increased cortical porosity and multiple spontaneous fractures in the hindlimbs.^30^ When pups grew to 3 and 6 weeks old, compared to their control littermates, *Piezo1*^*Prx1*^ mice exhibited significantly shorter and smaller long bones with decreased cortical and trabecular bone mass.^29,30^ These results suggest that Piezo1 contributes minimally to skeletal patterning but is critical for bone formation during skeletal development. These results also suggest that the abnormal bone phenotype from Piezo1 deficiency may be associated with mechanical loading. During pregnancy, embryos float in an amniotic fluid environment in the uterus, which results in less weight bearing for embryonic bones.^30^ However, after birth, the weight-bearing long bones, especially the hindlimbs, started to show defects.

**Table 1:** Piezo1 in bone development and homeostasis

	Cre	Development		Bone phenotype			Loading model	
		Embryo and Newborn	Young adult	Overall	Bone formation	Bone resorption	Loading model	Unloading model
MSC	*Prx1-Cre*^30^	P0: subtle difference P3: increased cortical porosity in femurs	6 weeks: smaller long bones; indistinguishable calvariae; decreased cortical and trabecular bone mass and bone surface	Short long bones; significant trabecular bone mass loss; reduced cortical bone thickness and surface; reduced collagen expression	No significantly affected stem cells and progenitors; no difference for osteoblastogenesis; no difference of serum PINP	Increased osteoclast number and bone resorption activities: increased eroded surface, increased osteoclast number	Under FSS, WT BMSC-derived osteoblasts strongly increase Col2α1 and Col9α2 expression but not in Piezo1-deficient cells	HLU did not induce bone loss in cKO mice; increased TRAP-positive cells in WT but not in cKO mice
	*Prx1-Cre*^29^	P0: multiple bone fractures in radius and ulna	3 weeks: short femurs	Short long bones; reduced cortical and trabecular bone mass	Reduced MAR, BFR; reduced osteoblast differentiation; reduced serum PINP; increase apoptosis in bone	Upregulated osteoclast differentiation: increased expression of osteoclast markers (Ctsk, TRAP)	n.a.	n.a.
Osteoblast lineage cell	*Osx-Cre*^29^	n.a.	3 weeks: multiple bone fractures in the ribs	Reduced trabecular and cortical bone mass	Reduced Osterix expression	n.a.	n.a.	n.a.
	*Runx2-Cre*^93^	P1: no calvarial bone defects; no datable trabecular bone loss P5: first rib fracture appears	2 weeks: multiple fractures in the ribs and femurs; shorter long bones. 12 weeks: pelvic dysplasia; reduced trabecular bone mass; no alteration of calvarial thickness or porosity	Enlarged hypertrophic zone at growth plate; remarkable reduction of trabecular bone mass below the growth plates	Moderate reduction in the mineral apposition rate at the trabecular bone surface; reduced serum P1NP and P1CP; altered flatten osteoblast morphology	Increased osteoclast number	n.a.	n.a.
	*Col2a1-Cre*^93^	n.a.	12 weeks: pelvic dysplasia; modulation of reduced bone length	Reduced trabecular bone volume with most pronounced in the secondary spongiosa; no significant reduction of cortical thickness	Increased flattened osteoblasts	n.a.	n.a.	n.a.
	*Col1-CreERT*^30^	n.a.	8-week aged mice, inject Tamoxifen for 2 weeks	Reduced trabecular bone mass and cortical thickness; compromised collagen expression	n.a.	Increased TRAP-positive osteoclasts	n.a.	n.a.
	*Ocn-Cre*^28^	P0: similar skeletal size, incomplete of cranial closure	8 and 16 weeks: shorter stature, lower body weight; significant bone loss in both male and female.	Shorter long bones; reduced long bone strength; loss of bone mass.	Reduced bone formation rate; reduced osteoblastic marker gene expression (Col1a1, Ocn, Bglap); reduced serum Ocn and PINP.	Similar TRAP straining; no change of Nfact1/Acp5/Ctsk/Mmp9 mRNA level; no change of serum CTX-1	21 days’ treadmill exercise: no significant increase of osteoblast genes in cKO mice	28 days’ HLU: no further reduction of bone mass or bone strength in cKO mice
	*Dmp1-Cre*^27^	Normal body weight	5, 8, and 12 weeks: normal body weight; low bone mineral density; difference increased as mice mature	Normal femur length; reduced trabecular bone mass and bone stiffness; spontaneous fractures in the tibia at 12 weeks	Reduced BFR; low osteoblast number; normal osteoblastogenesis; reduced Wnt1 mRNA in cortical bone; unaffected Sost expression in cortical bone	Increased osteoclast number; increased pro-osteoclastogenic cytokine RANKL but no changes for expression of OPG	14 days’ tibia loading: blunted load-stimulated bone formation in cKO mice	n.a.
	*Dmp1-Cre*^30^	n.a.	n.a.	Decreased bone mass in both trabecular and cortical bones; no spontaneous fractures	n.a.	Increased osteoclast numbers	n.a.	n.a.
	*Dmp1-Cre*^93^	n.a.	12 weeks: reduced trabecular and cortical bone mass	No significant changes in osteocyte number or the number of canalicular extensions	Altered trabecular osteoblasts with a flattened appearance in cKO mice	n.a.	Three consecutive days of ulna loading: reduced bone formation compared to control mice	n.a.
Osteoclasts	*Ctsk-Cre*^30^	n.a.	n.a.	Normal bone mass	n.a.	Unaffected bone resorption	n.a.	n.a.
	*Lyz2-Cre*^93^	n.a.	12 weeks: no detectable skeletal phenotype	n.a.	n.a.	n.a.	n.a.	n.a.

*P* postnatal, *PINP* aminoterminal propeptide of type I collagen, *FSS* fluid shear stress, *WT* Wield type, *BMSC* bone marrow stromal cell, *Col* collagen, *HLU* hind limb unloading, *Ctsk* Cathepsin K, *TRAP* tartrate-resistant acid phosphatase, *cKO* conditional knockout, *MAR* mineralization apposition rate, *BFR* bone formation rate, *Ocn* osteocalcin, *Bglap* bone gamma-carboxyglutamate protein, *Nfact1* nuclear factor kB activator 1, *Acp5* acid phosphatase 5, *Mmp9* matrix metallopeptidase 9, *CTX-1* crosslinked C-telopeptide of type I collagen, *Wnt1* Wnt family member 1, *Sost* sclerostin, *OPG* osteoprotegerin, *n.a.* not applicable

Bone modeling is tightly controlled by a balance between bone formation and bone resorption. To unveil the mechanisms underlying the dramatic bone loss in *Piezo1*^*Prx1*^ mice, the cellular activities of both bone formation and bone resorption were examined. Wang et al. did not observe significant changes in osteoblastogenesis or the serum levels of procollagen type I N-terminal propeptide (P1NP), an in vivo marker for bone formation, in *Piezo1*^*Prx1*^ mice.^30^ In contrast, Zhou et al. observed reduced bone formation, reduced serum levels of P1NP, and decreased expression of Osterix, an osteoblast differentiation marker, in *Piezo1*^*Prx1*^ bone samples.^29^ The reasons for this discrepancy remain to be determined. Despite the contradictory observations on bone formation, the bone loss in *Piezo1*^*Prx1*^ mice was confirmed by increased bone resorption in the Piezo1-deficient mice. Both research groups reported that *Piezo1*^*Prx1*^ mice exhibited increased osteoclast number and osteoclast differentiation, accompanied by enhanced bone-resorbing activities at 3 and 6 weeks of age.^29,30^

Interestingly, the low bone mass phenotype in *Piezo1*^*Prx1*^ mice seems to be restricted to load-bearing long bones. The calvariae, which are less load-bearing than long bones,^95^ from *Piezo1*^*Prx1*^ mice were indistinguishable from their control littermates at 6 weeks of age.^30^ To test the involvement of Piezo1 from MSCs in mechanotransduction, both control and *Piezo1*^*Prx1*^ mice were subjected to 6 days of tail suspension. The results showed that mechanical unloading from tail suspension led to bone mass loss in control mice but not in *Piezo1*^*Prx1*^ mice.^30^ This difference resulted from nonresponsive osteoclasts in Peizo1 deletion mice. Recently, Piezo1 from Osteolectin^+^ cells, a subset of skeletal stem cells and progenitors in bone marrow, was reported to have essential functions in maintaining bone mass.^96^ Mice with Piezo1 deletion in Osteolectin^+^ cells displayed reduced bone mineral density and cortical bone thickness at 2 months of age.^96^ Together, these results demonstrate the importance of Piezo1 in MSCs and progenitor cells for mechanotransduction during bone development through inhibiting osteoclast-mediated bone resorption.

During skeletal development, MSCs differentiate into osteoblast lineage cells, which are controlled by several key transcription factors. Among these factors, Runt-related transcription factor 2 (Runx2) is a master regulator that controls the commitment of MSCs to osteoblastic lineage cells during bone development.^97,98^ Osterix is actively expressed in osteoblast progenitors and osteoblasts and is considered a preosteoblast marker.^99,100^ Collagen type 1 (Col1) is expressed from preosteoblasts to mature osteoblasts.^101^ Moreover, osteocalcin is highly expressed in mature osteoblasts,^100^ and Dmp1 is a well-known marker for osteocytes and mature osteoblasts.^102,103^ To examine the involvement of Piezo1 at different osteoblastic development stages, different promoters of these transcription factors were used in transgenic mouse models.

Mice lacking Piezo1 in Runx2-expressing cells (*Piezo1*^*Runx2*^)^93^ showed normal calvarial bone with no marked trabecular bone mass change in the vertebral body at P0.^93^ However, the first occurrence of rib fracture appeared at P5, and limb fractures appeared at 2 weeks of age in all *Piezo1*^*Runx2*^ mice.^93^ Moreover, *Piezo1*^*Runx2*^ mice of both sexes displayed shortening of the long bones and pelvic dysplasia.^93^ Along with these observations, a remarkable reduction in trabecular bone mass was observed from 2 weeks of age onwards.^93^ These bone defects were tightly associated with abnormal osteoblast functions in these animals, which presented a flattened appearance of osteoblasts on the surface of trabecular bone and significant reductions in serum P1NP and procollagen type I carboxyterminal propeptide levels in *Piezo1*^*Runx2*^ mice.^93^

The deletion of Piezo1 in Osterix-positive cells (*Piezo1*^*Osx*^) caused multiple bone fractures in the ribs in 3-week-old mice without causing any obvious bone defects at P0.^29^ *Piezo1*^*Osx*^ mice showed a reduction in the expression of Osterix and trabecular and cortical bone mass.^29^ The inducible deletion of Piezo1 in osteoblasts with *Col1-Cre/ERT* (*Piezo1*^*Col1ERT*^) in 10-week-old mice also resulted in reduced trabecular bone mass, decreased cortical thickness, and compromised collagen expression.^30^ *Piezo1*^*Col1ERT*^ mice showed increased bone resorption, as demonstrated by more tartrate-resistant acid phosphatase-positive osteoclasts.^30^ Another study was conducted for Piezo1 deletion in mature osteoblasts using mouse *Osteocalcin-Cre* (*Piezo1*^*Ocn*^).^28^ At P0, *Piezo1*^*Ocn*^ mice displayed skeletal sizes similar to those of their control littermates, but the mutant mice had incomplete closure of their cranial sutures.^28^ At 8 and 16 weeks of age, compared to control littermates, *Piezo1*^*Ocn*^ mice suffered from significant bone mass loss, shorter weight-bearing long bones (femurs and tibiae), and reduced long bone strength, resulting in shorter stature and lower body weight.^28^ Cellular- and tissue-level examinations further revealed a significant reduction in osteoblast differentiation in the bone of *Piezo1*^*Ocn*^ mice.^28^ Consistent with these phenotypes, both mechanical loading (treadmill exercise) and unloading (hindlimb unloading model) failed to alter osteoblast and osteoclast functions in *Piezo1*^*Ocn*^ mice.^28^ Therefore, these results demonstrate that Piezo1 expression in osteoblasts contributes to mechanosensation under both load and unloading conditions, which is essential for proper bone growth in development and homeostasis.

**Fig. 2:**
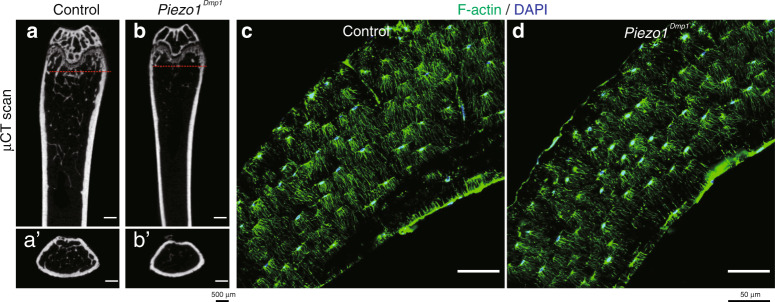
Osteocyte Piezo1 deficiency leads to significant bone loss. **a**, **b** Micro-CT scanning of distal femurs from 3-month-old control (*Piezo1*^*fl/fl*^) and conditional knockout (cKO) mice with specific Piezo1 loss in osteocytes (*Piezo1*^*Dmp1*^). **a’**, **b’** Cross-section of CT scan images at the red line in **a**, **b** for trabecular and cortical bone mass detection in Control and cKO mice. **c**, **d** Rhodamine-phalloidin staining of the F-actin cytoskeleton of cross-section samples of femurs from 3-month-old control and cKO mice. F-actin in green; DAPI in blue

At the final stage of osteoblast differentiation, the majority of differentiated osteoblasts become osteocytes that are embedded in the mineralized matrix. Osteocytes are considered master regulators and major mechanoresponsive cells in bone remodeling.^79–81,90^ Deletion of Piezo1 using *Dmp1-Cre* transgenic mice resulted in progressive osteopenia^29,30,93^ (Fig. 2). *Piezo1*^*Dmp1*^ mice had normal body weights from newborns (P0) to young adults (12 weeks).^27,30^ Although *Piezo1*^*Dmp1*^ mice had normal femur length, microcomputerized tomography revealed that *Piezo1*^*Dmp1*^ mice displayed significantly reduced trabecular bone mass and bone stiffness.^27,30^ Li et al. observed spontaneous fractures in the tibiae of *Piezo1*^*Dmp1*^ mice at 12 weeks of age,^27^ whereas Wang et al. reported no spontaneous fracture in their *Piezo1*^*Dmp1*^ mice.^30^ This difference could be explained by the different *Dmp1* promoters used in the studies: Li et al. used the 8-kb *Dmp1-Cre*, while Wang et al. used the 9.6-kb *Dmp1-Cre*. The osteopenic phenotypes raised from osteocyte Piezo1 deletion could result from both reduced bone formation and enhanced bone resorption. Li et al. reported a reduced bone formation rate in *Piezo1*^*Dmp1*^ mice, which is further supported by low osteoblast number and reduced *Wnt1* mRNA expression in cortical bone.^27^ Increased osteoclast number and enhanced pro-osteoclastogenic cytokine Rankl expression were observed in *Piezo1*^*Dmp1*^ mice.^27^ Anabolic tibial loading^27^ and ulna loading^93^ experiments further showed that *Piezo1*^*Dmp1*^ mice failed to thicken their cortical bone due to impaired bone formation. In short, Piezo1 in osteocytes regulates mechanotransduction under load conditions by activating bone formation and inhibiting bone resorption.

In addition to the osteoblast lineage cells discussed above, Wang et al. deleted Piezo1 expression in osteoclasts through *Ctsk-Cre* in mice.^30^ *Piezo1*^*Ctsk*^ mice developed normally with indistinguishable stature and body weight, normal bone mass, and unaffected bone resorption compared to their control littermates.^30^ These results were consistent with the low expression of Piezo1 in the osteoclast-like cell line RAW264.7.^28^ Similarly, mice with osteoclast deletion of Piezo1 generated by Hendrickx et al. using *Lyz2-Cre* displayed a normal skeletal phenotype.^93^

Compared to Piezo1, the phenotype of Piezo2 deficiency in osteoblast lineage cells is trivial. Deletion of *Piezo2* in MSCs with *Prx1-Cre* or in osteoblast lineage cells with *Osterix-Cre* displayed grossly normal skeletal development at P0 and no obvious length difference in long bones with normal bone mass at later stages.^29^ Unlike Piezo1 deletion in these cells, loss of Piezo2 caused no fractures in mice.^29^ Considering the low expression of Piezo2 in osteoblasts and osteocytes,^27,92^ these observations suggest that Piezo2 alone has limited contributions to bone development and bone homeostasis. Interestingly, when Piezo1 and Piezo2 were doubly deleted (dKO) in MSCs, more severe skeletal defects were observed than Piezo1 single-KO.^29^ In dKO mice, additional fractures were found in the femurs at P0 compared to the forelimb fractures observed in Piezo1 single-KO mice.^29^ dKO mice also had shorter long bones and more severely reduced cortical and trabecular bone mass than Piezo1 single-KO mice.^29^ At the tissue level, dKO mice showed further reductions in the bone formation rate, expression of Osterix, and serum level of P1NP, as well as further upregulation of the expression of osteoclast marker genes, compared to Piezo1 single-KO mice.^29^ In addition, dKO bone showed enhanced cell apoptosis.^29^ This aggravating effect on bone phenotypes was also observed in Piezo1- and Piezo2-dKO mice using *Runx2-Cre*^93^ or *Osterix-Cre*^29^ but not *Dmp1-Cre.*^93^ Collectively, these results suggest an important role of Piezo1 in osteoblast lineage cells and a functional redundancy of Piezo1 and Piezo2 in bone.

In addition, several pioneering studies have been conducted to manipulate Piezo activation in a mouse model and cell culture. Li et al.^27^ tested the Piezo1 agonist Yoda1 in WT mice. Yoda1 administration to 4-month-old female WT C57BL/6J mice for 2 weeks at 5 μmol·kg^−1^ body weight significantly increased bone mass in the treatment group without obvious effects on body weight or osteoclast bone resorption activity.^27^ Zhang and co-workers reported that low-intensity pulsed ultrasound (LIPUS) stimulated Piezo1 channels in the osteoblastic cell line MC3T3-E1.^104^ LIPUS-induced Piezo1 channel opening allowed calcium influx, activated extracellular signal–regulated kinase 1/2 (ERK1/2) phosphorylation and F-actin polymerization, and further enhanced cell migration and proliferation.^104^ The authors proposed a potential mechanism: PIEZO1 activation in osteoblasts is tightly related to LIPUS-induced bone regeneration and fracture repair in clinical practice.

Taken together, these genetic mouse studies demonstrate a critical role for Piezo1 in MSCs and osteoblast lineage cells in the regulation and maintenance of skeletal development and homeostasis. Piezo1 mediates cellular mechanosensation in MSCs, osteoblasts, and osteocytes, which further influences bone formation and bone resorption. Furthermore, Piezo2 acts as an additional partner for Piezo1 in the control of bone mass and mechanotransduction.

## Signaling pathways related to Piezo1 in bone

**Fig. 3:**
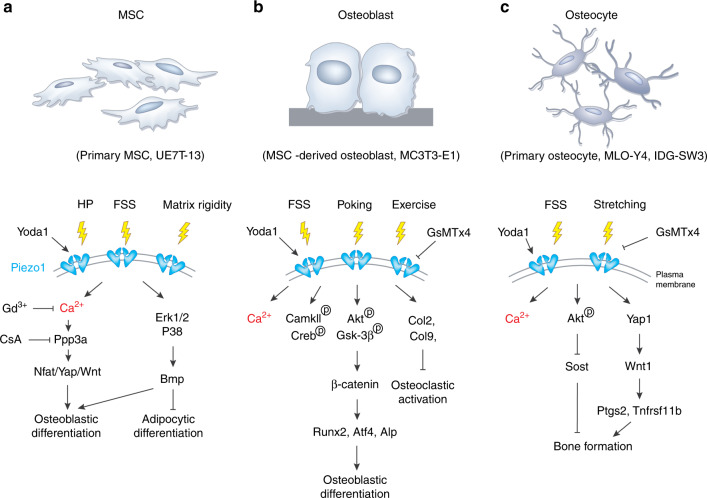
Piezo1 signaling in osteoblast lineage cells. **a** The activation of Piezo1 channels in primary MSCs or UE7T-13 cell lines can be triggered by hydrostatic pressure (HP), fluid shear stress (FSS), matrix rigidity, and Yoda1, which further activate the Ppp3a-Nfat/Yap-Wnt pathway through Ca^2+^ signals and Bmp signals through the Erk1/2-p38 pathways. The activation of Piezo1 in MSCs leads to osteoblastic differentiation and inhibition of adipocytic differentiation. **b** Mechanical stimuli, such as FSS, poking, and exercise, induce Piezo1 activation in MSC-derived osteoblasts and MC3TC-E1 cell lines. Piezo1 activation stimulates intracellular signal responses, including Ca^2+^ flux, CamKII-Creb signaling, Akt-Gsk3β signaling, and collagen type 2 and 9 expression in osteoblasts. As a result, Piezo1 activation enhances osteoblastic differentiation but inhibits osteoclastic activation. **c** Piezo1 channels from primary osteocytes, MLO-Y4 or IDG-SW3 cell lines can be triggered by FSS and stretching stimuli. The activation of Piezo1 further induces Ca^2+^ flux, phosphorylation of Akt, and Yap1 activation in osteocytes. As a result, Piezo1 channels contribute to bone formation in these cells

Consistent with its in vivo gene expression, Piezo1 is highly expressed in cell lines and primary cells derived from MSCs and osteoblast lineage cells in vitro. Experiments conducted on cultured cell systems unveil several important signaling pathways that are involved in the mechanical activation and regulation of Piezo1 channels in bone (Fig. 3).

Piezo1 is expressed in both primary MSC and MSC-derived cell lines. Published studies demonstrate an essential function of Piezo1 in MSC fate determination under various mechanical stimuli.^29,105^ In cultured MSCs, the expression of Piezo1 was localized to the plasma membrane of human bone marrow-derived UE7T-13 cells (human MSCs) with especially high accumulation in cellular lamellipodia and filopodia tips.^105^ The results of hydrostatic pressure (HP) stimulation showed that 0.01 MPa HP induced the expression of Piezo1 but not that of Piezo2 or transient receptor potential vanilloid 4 (TRPV4) in UE7T-13 cells.^105^ HP or Yoda1 induced the expression of bone morphogenetic protein 2 (Bmp2) in UE7T-13 cells. The upregulation of Bmp2 seems to be mediated by Erk1/2 and p38, whose phosphorylation is modulated by Piezo1. Since Bmp2 is an essential bone morphogenetic protein that promotes the differentiation of MSCs into osteoblasts,^106^ this Piezo1-Bmp2 pathway controls MSC stem cell fate by promoting osteoblast differentiation and inhibiting adipocyte differentiation during bone development. In addition to HP stimulation, cultured primary MSCs displayed more robust Ca^2+^ influx and enhanced osteogenic differentiation when they were subjected to fluid shear stress (FSS).^29^ However, these processes were blocked by the Cre adenovirus-mediated deletion of Piezo1 in MSCs.^29^ The deletion of Piezo1 in MSCs abolished their osteogenic responses to FSS due to inactivation of Yes associated protein 1 (Yap1) and catenin β1 (Ctnnb1) signaling by increasing their phosphorylation for cytoplasmic degradation.^29^ The results from in vitro bone marrow stromal cell (BMSC) culture and in vivo bone samples further suggest that Piezo1-dependent Yap1 and Ctnnb1 activation is mediated by nuclear factor of activated T cells (Nfat) activation through protein phosphatase 3 catalytic subunit alpha (Ppp3ca), an important intracellular Ca^2+^ sensor in cells.^29^ Piezo1-activated Ppp3ca/Nfat/Yap1/Ctnbb1 signaling was enhanced by Yoda1 treatment and inhibited by gadolinium (Gd^3+^, a potent calcium channel blocker) and cyclosporin A (a commonly used Ppp3ca inhibitor).^29^ Furthermore, BMSCs can sense substrate rigidity and translocate cytoplasmic Yap1 to the nuclear compartment when cultured on a stiff matrix (40 kPa).^29^ Piezo1-deficient BMSCs lost the ability to sense and respond to stiff substrates and displayed weaker intracellular Ca^2+^ signaling with diffusive Yap1 localization in the cytoplasm and nuclei.^29^

*Piezo1* mRNA was also expressed in murine osteoblastic MC3T3-E1 cells^105,107^ and primary BMSC-derived osteoblasts.^28^ The expression level of Piezo1 is tightly associated with osteoblastic differentiation capacity. In an in vitro osteogenic medium-induced differentiation model, higher expression of Piezo1 was detected in more mature osteoblasts.^28^ Knockdown of Piezo1 expression by siRNA in MC3T3-E1 cells decreased the expression of osteoblastic marker genes, including alkaline phosphatase (Alp), osteocalcin, and collagen type 1 alpha 1 (Col1a1).^28^ Piezo1 in osteoblasts also actively participates in mechanical stimuli-induced calcium flux. Piezo1 protein expression was increased in MC3TC-E1 cells by FSS in a timely manner.^107^ There was a 2.6-fold increase in Piezo1 expression after 30 min of FSS treatment, but the difference disappeared within 60 min after treatment.^107^ The activation of Piezo1 upon FSS stimuli further induced the phosphorylation of protein kinase B (PKB/AKT) and GSK-3β, followed by the translocation of β-catenin into the nuclei, where it modulated Runx2 expression.^107^ Reducing Piezo1 expression by siRNA or pharmacologically inhibiting Piezo1 through GsMTX4 greatly reduced the MA currents in MC3T3-E1 cells.^28^

In addition to osteoblastic cell lines, primary osteoblasts need Piezo1 for mechanotransduction. Primary osteoblasts showed significantly increased expression of Piezo1 and osteoblast marker genes upon FSS treatment for 2 h in culture.^28^ However, these cells had dramatically reduced expression of osteoblast marker genes and Alp activity in a cell rotation system that generated a microgravity condition.^28^ Furthermore, primary osteoblasts derived from *Piezo1*^*Ocn*^ mice exhibited significantly reduced poking- or Yoda1-induced currents with drastically reduced Ca^2+^ staining.^28^ Molecularly, osteoblasts from *Piezo1*^*Ocn*^ mice had decreased phosphorylation of CaMKII and Creb and reduced expression of Runx2 and Atf4, two key transcription factors required for osteoblast differentiation.^28^ Interestingly, osteoblasts from *Piezo1*^*Ocn*^ mice treated under microgravity through cell rotation decreased MA currents.^28^ In addition, BMSC-derived osteoblasts from *Piezo1*^*Prx1*^ mice displayed a global transcriptome difference from wild-type (WT) osteoblasts.^30^ Specifically, in Gene Ontology analysis, the expression of genes encoding the extracellular matrix (ECM) proteins Col2α1, Col9α1/2, and Col10α1 was significantly downregulated by Piezo1 deletion.^30^ Interestingly, the expression levels of Col2α1 and Col9α1 were strongly induced by FSS treatment in WT osteoblasts but not in osteoblasts derived from *Piezo1*^*Prx1*^ mice. Since Col2^108^ and Col9^109^ were reported to inhibit osteoclast activity, these results could explain the bone mass loss in *Piezo1*^*Prx1*^ mice through enhanced osteoclast activities.^30^

Piezo1 was also expressed in the osteocyte cell lines IDG-SW3^110^ and MLO-Y4.^27^ The results showed that, among 78 calcium channels detected in MLO-Y4 cells under static conditions, Piezo1 was the most highly expressed calcium channel.^27^ Specifically, Piezo1 displayed an approximately 200-fold higher expression level than Piezo2 in MLO-Y4 cells.^27^ Published studies demonstrate an essential role of Piezo1 in regulating mechanical stimuli responses in osteocytes. In IDG-SW3 cells, mechanical cyclic stretching through cell culture substrates inhibited Sclerostin expression by phosphorylating PKB/AKT at the Ser473 site.^110^ This process was inhibited by the pharmacological inhibition of Piezo1 by GSMTx4 or by Piezo1 deletion.^110^ In MLO-Y4 cells, the results from both RNA sequencing and real-time quantitative PCR (RT–qPCR) analyses showed that FSS significantly upregulated the expression of Piezo1.^27^ Downregulation of Piezo1 by short hairpin RNA (shRNA) largely blocked the increases in intercellular calcium and the expression of Ptgs2 (prostaglandin-endoperoxide synthase 2) and Tnfrsf11b (TNF receptor superfamily member 11b) induced by FSS.^27^ Silencing Piezo1 in MLO-Y4 cells blunted Wnt1 expression and Yap1 activation by FSS.^27^ Piezo1 overexpression or Yoda1-induced Piezo1 activation further enhanced the expression of Ptgs2 and Tnfrsf11b under FSS treatment.^27^ To further evaluate Piezo1 activation in the bone formation process, Yoda1 was administered to 4-month-old C57BL/6J mice for 2 weeks.^27^ Examination of bone samples showed increases in cortical bone thickness and cancellous bone mass in the distal femur after Yoda1 treatment, without any obvious alteration in mouse body weight.^27^ This bone formation induced by Yoda1-activated Piezo1 channels resulted from increased bone formation, which was confirmed by the increased serum level of osteocalcin in Yoda1-treated mice.^27^ In addition to cell lines, primary osteocytes derived from *Piezo1*^*Ocn*^ mice exhibited significantly reduced poking-induced currents compared to control osteocytes.^28^ Moreover, Sost expression was significantly upregulated in bone tissue from *Piezo1*^*Ocn*^ mice.^28^ Together, these results demonstrate the importance of Piezo1 in the mechanotransduction of MSCs and osteoblast lineage cells.

## Piezo proteins in chondrocytes

Articular cartilage is another important part of the skeleton that supports mechanical loading from body weight and daily activities. Articular cartilage is the hydrated connective tissue that lines the contacting surfaces of the opposing bones in diarthrodial joints, whose function is to minimize friction and distribute mechanical loading within the joints.^111,112^ This cartilage tissue is avascular and consists primarily of chondrocytes and ECM.^113,114^ Chondrocytes are the only cells in articular cartilage and can sense a complex array of mechanical stimuli, including stretching force, compression loading, and shear stress within the joints through molecules and receptors resident in the cell membrane, such as integrins, Ca^2+^ channels, and primary cilia.^115–118^ These mechanoreceptors transduce mechanical signals into biochemical signals, modulating the balance between anabolism and catabolism of the ECM in cartilage homeostasis.^85,119^ In a pathological scenario, altered mechanical loading patterns could lead to the onset and progression of osteoarthritis (OA) due to the degradation of articular cartilage raised from faulted mechanotransduction in chondrocytes.^120,121^ Recently, an increasing number of studies have focused on cellular and molecular mechanotransduction signaling in chondrocytes.

Piezo proteins have been demonstrated to have robust expression in chondrocytes and play a vital role in the mechanotransduction of chondrocytes.^85,122^ In 2014, Lee et al.^123^ performed experiments to examine the expression of Piezo channels in articular cartilage of humans, pigs, and mice by RT–qPCR analysis and immunofluorescence staining. They found that both Piezo1 and Piezo2 were highly expressed in primary chondrocyte cultures and cartilage tissues. Du et al.^124^ also observed appreciable expression of both Piezo1 and Piezo2 proteins in mouse primary chondrocytes by western blotting. However, in contrast to the above results, Servin-Vences et al.^121^ reported that the Piezo1 transcript, but not the Piezo2 transcript, could be reliably measured in mouse primary chondrocytes by RT–qPCR analysis. In addition, an animal study showed that loss of Piezo1 in chondrocytes with *Col2a1-Cre* (*Piezo1*^*Col2a1*^) led to a high incidence of skeletal fractures and pelvic dysplasia in mice at 12 weeks of age.^93^ *Piezo1*^*Col2a1*^ mice had no obvious defect in cortical bone thickness but displayed a nearly 50% reduction in trabecular bone volume compared to their age-matched control littermates.^93^ Interestingly, the defects were most pronounced in the secondary spongiosa, where large numbers of flattened osteoblasts were found on the *Piezo1*^*Col2a1*^ trabecular bone surface.^93^ These data suggest that Piezo1 is essential for endochondral ossification. More detailed studies are required to examine the expression of Piezo2 in chondrocytes.

**Fig. 4:**
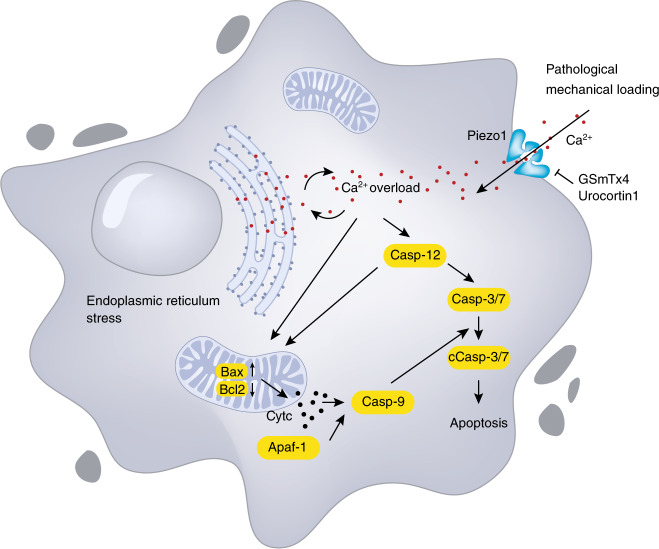
Piezo1 signaling in chondrocytes. Under pathological mechanical loading, the expression of Piezo1 protein is increased, resulting in excessive Ca^2+^ influx. Ca^2+^ overload activates endoplasmic reticulum (ER) stress and upregulates the expression of caspase-12 (Casp-12), leading to the expression of caspase-3/7 (Casp-3/7), Bax, and Bcl2 and the release of cytochrome c (Cytc). Cytc and Apaf-1 upregulate the expression of caspase-9 (Casp-9), which activates Casp-3/7 to cleave Casp-3/7 (cCasp-3/7) and finally results in chondrocyte apoptosis. GsMTx4 and urocortin1 can exert chondroprotective effects by inhibiting Piezo1 and preventing Ca^2+^ overload

Published data suggest that Piezo channels mediate mechanotransduction in chondrocytes mainly through their regulation of Ca^2+^ influx.^119^ Du and co-workers demonstrated that high levels of cyclic tensile strain (CTS) could upregulate the expression of Piezo1 and Piezo2 in chondrocytes.^124^ Moreover, compressing isolated porcine chondrocytes with atomic force microscopy at high levels of compression (>45% strain) significantly increased the intracellular Ca^2+^ levels.^123^ This calcium influx could be suppressed by gene modification through Piezo1 or Piezo2 siRNA or by pharmacological treatment with a Piezo1 inhibitor GsMTx4.^123^ Furthermore, [Ca^2+^]_*I*_ responses in isolated mouse chondrocytes were significantly evoked by CTS stimulation at a high strain level of 18%, which was obviously inhibited by Piezo1 or Piezo2 knockdown.^124^ It should be noted that these mechanical treatments mentioned above are considered to be hyperphysiological and injurious loadings, which can lead to the development of OA.^122^ Therefore, the elevated Piezo activation upon high-strain mechanical stimuli in chondrocytes could play a crucial role in promoting the initiation and progression of OA. Cumulative evidence from cultured chondrocytes supports this notion. In cultured human chondrocytes, abnormal mechanical stretch force increased the expression of PIEZO1 protein, resulting in excessive Ca^2+^ influx. Massive cytoplasmic Ca^2+^ activated ER stress and upregulated the expression of caspase-12, which further led to chondrocyte apoptosis by activating the mitochondrial pathway (Fig. 4).^125^ Since cell death is an important pathological feature of OA,^126^ it is possible that blocking Piezo1 activity provides some protective effects for chondrocytes. Reported results showed that Piezo1 inhibition by GsMTx4 could protect chondrocytes against high strain-induced cell death.^123^ Moreover, urocortin1, a corticotropin-releasing factor-related peptide in chondrocytes, was found to exert chondroprotective effects by maintaining Piezo1 in a closed conformation and preventing Ca^2+^ overload.^127^

In addition to calcium flux, Piezo1 participates in OA pathogenesis, probably by promoting inflammatory responses in chondrocytes.^128^ In human samples, osteoarthritic cartilage had significantly increased Piezo1 expression compared to healthy cartilage.^128^ Lee and colleagues recently showed that interleukin-1α (IL-1α), an OA-related inflammatory cytokine, upregulated Piezo1 expression, and abnormal F-actin cytoskeleton patterns in porcine chondrocytes.^128^ These chondrocytes became more sensitive to injurious levels of loading, exhibiting enhanced Ca^2+^ signaling upon Yoda-1 treatment or mechanical loading.^128^ Mechanistically, IL-1α enhanced Piezo1 expression through p38 mitogen-activated protein kinase and transcription factors HNF4 and ATF2/CREBP1 in chondrocytes, where CREBP1 has been shown to bind directly to the proximal *PIEZO1* gene promoter.^128^ This whole process is considered a positive feedback loop in which IL-1α exposure leads to Piezo1 upregulation, which further enhances the sensitivity of chondrocytes in response to external loading and finally the progression of OA.^128^

Moreover, one essential issue regarding OA is joint pain upon sustained weight bearing and joint movement. Published data suggest that blocking nerve growth factor activities reduced joint pain and improved joint functions in OA patients^129^ and that OA pain partially resulted from sensory neuron axonal growth in subchondral bone by osteoclast secretion of nerve growth factor netrin-1.^130^ These results suggest that sensory innervation is tightly associated with OA pain. Considering the extensive involvement of neuronal Piezo2 in skeletal homeostasis,^131^ it would be worth examining the function of Piezo2 in OA pain. Thus, developing drugs or inhibitors targeting Piezo proteins could be beneficial to chondrocyte survival as a potential treatment for OA. Taken together, current data support that Piezo proteins play an essential role in chondrocyte mechanotransduction and may serve as a novel target for OA therapy.

## Piezo proteins in other cells and tissues

Piezo proteins are highly expressed in various mammalian cells and tissues, including neurons, myeloid cells, adipocytes, lungs, blood vessels, smooth muscle, guts, bladder, and skin. Regarding the diverse functions of Piezo proteins in different tissues, we reviewed and summarized the phenotypes from the tissue-specific loss of *Piezo1* and/or *Piezo2* in experimental transgenic mouse models (Tables 2 and 3).

**Table 2:** Conditional deletion of Piezo1 in experimental mice

Tissue type	Cre	Phenotype	Mechanism
Global deletion	^63^	Embryos died at E9.5 lacking modeling of vasculature	Impaired endothelial cell alignment in response to shear stress; failing to remodel arteries
Endothelial cells	*Cadherin5-CreERT2*^181^	Inhibited Ca^2+^ influx induced by Yoda1; more sensitive to α-adrenergic agonists	
	*Cadherin5-CreERT2*^137^	Impaired physical performance and lower body weight after sustained activity	Piezo1 transduced fluid flow signal to constriction of mesenteric arteries, which are responsible for total peripheral resistance
	*iEC-Cre*^132^	Increased lung vascular permeability by high-volume mechanical ventilation	Piezo1 targeted calcium-dependent cysteine protease calpain to maintain homeostasis of the endothelial barrier
	*iEC-Cre*^133^	Increased lung microvessel pressure; impaired regulation of lung endothelial barrier	Piezo1 regulated lung vascular permeability by targeting endothelial VE-cadherin
	*Tie2-Cre or Lyve1-Cre*^134^	Reduced amount of lymphatic valves	
	*Tie2-CreERT2*^138^	Upregulated arterial blood pressure	Failed to produce NO or vasodilate because of insensitivity to flow stimulation
Adult endothelial cells	*SCL-CreERT*^136^	Impaired angiogenesis; inhibited endothelial sprouting and lumen formation after subjected to wall shear stress	Piezo1 increased intracellular Ca^2+^ and activated MT1-MMP pathway
Lymphatic endothelial cells	*Prox1-CreERT2*^135^	Inhibited formation and maintenance of lymphatic valves and lymphatic vessels	Piezo1 transduced the signal of OSS to control lymphatic valves and lymphatic vessels
Smooth muscle cells	*sm22-Cre*^14^	Reduced activity of ion channels in caudal artery myocytes induced by stretch; reduced arterial diameter, wall thickness, and cross-sectional area treated with Angiotensin II	Piezo1 sensed flow and pressure to regulate structural remodeling of arteries
Adipocytes	*Adiponectin-Cre*^141^	Increased insulin resistance; decreased pgWAT weight and increased pro-inflammatory and lipolysis genes after HFD fed; hepatic steatosis with increased fatty acid synthesis genes	Piezo1 participated in TLR4-mediated inflammation
	*Adiponectin-CreERT2*^140^	Impaired adipocyte differentiation, causing inflammation and insulin insensitivity	Piezo1 promoted the secretion of adipogenic FGF1 to facilitate adipocyte precursor differentiation
Myeloid cells	*LysM-Cre*^143^	Ameliorated pulmonary inflammation	Piezo1 exhibited proinflammatory effects by activating AP-1c and EDN1 and stabilizing HIF1α
Acinar cells	*Ptf1a-CreER*^142^	Ameliorated pancreatitis, including reduced edema, neutrophil infiltration, hemorrhage, and tissue necrosis	Piezo1 activated cytoplasmic calcium signals that are toxic to pancreatic acinar cells, resulting in cellular necrosis and pancreatitis
Nodose and petrosal sensory ganglia neurons	*Phox2b-Cre Piezo1/2 dKO*^156^	Attenuated baroreflex and activity of aortic depressor nerve	
DRG neurons	*shRNA KD*^159^	Inhibited Ca^2+^ influx induced by Yoda1	The mechanism is still unclear whether targeting Ca^2+^ -induced phospholipase C δ, β, or other signaling pathways

*E* embryonic, *VE* vascular endothelial, *NO* nitric oxide, *MT1* membrane-type 1, *MMP* matrix metalloproteinase, *OSS* oscillating shear stress, *pgWAT* perigonadal white adipose tissue, *HFD* high fat diet, *TLR4* Toll-like receptor 4, *FGF1* fibroblast growth factor 1, *AP-1* activator protein-1, *EDN1* endothelin-1, *HIF1α* hypoxia-inducible factor-1α, *DRG* dorsal root ganglion

**Table 3:** Conditional deletion of Piezo2 in experimental mice

Tissue type	Cre	Phenotype	Mechanism
Global deletion	^182^	Lethal within 24 h of birth	
CNS neurons	*AAV-Cre injection to Piezo2*^*fl/fl*^^149^	Insensitive to gentle dynamic touch but still sensitive to noxious pinch	
Mesencephalic trigeminal nucleus	*Pvalb-Cre*^154^	Diminished limb coordination	
Peripheral sensory neurons	*Advillin-Cre*^41^	Less sensitive to touch and proprioception but more sensitive to mechanical pain responses	Piezo2-mediated gentle touch sensation inhibited mechanical pain, indirectly mediating mechanical pain responses
	*Advillin-Cre*^153^	Reduced percentage of rapidly adapting neurons and of intermediately and slowly adapting neurons; reduced mechano-sensitive terminals and lower frequency of nerve terminal impulse discharges subjected to mechanical stimulation; decreased eye blinks evoked by von Frey filaments	Piezo2 was involved in the transduction of noxious mechanical forces by pure mechanosensory and polymodal nociceptor corneal neuron classes
	*Advillin -CreERT2*^94^	Impaired touch sensation	Loss of mechanically activated currents in DRG neuronal cultures
	*Advillin-CreERT2*^26^	Reduced vagal nerve firing in response to lung inflation; increased tidal volume	
Proprioceptive neurons	*Pvalb-Cre or HoxB8-Cre*^22^	Uncoordinated body movements and malposed limbs; reduced firing of proprioceptors in the muscle nerve induced by stretch	Piezo2-deficient proprioceptive neurons were most insensitive to mechanical signals
Na(v)1.8-positive sensory neurons	*Scn10a-Cre*^155^	Impaired bladder control and sensation of bladder filling; longer intervals between bladder contractions	Piezo2 regulated neuronal stretch responses, targeting bladder muscles and/or stretch-related cells
Nodose ganglia neurons	*Phox2b-Cre*^26^	No effect on oxygen saturation or lung structure	Piezo2 transduced the stretch of airway-innervating vagal neurons
Jugular, trigeminal, and DRG neurons	*Wnt1-Cre*^26^	Respiratory distress and smaller airspaces	
Nodose and petrosal sensory ganglia neurons	*Phox2b-Cre Piezo1/2 dKO*^156^	Attenuated baroreflex and activity of aortic depressor nerve	
Urothelial cells	*Upk2-Cre*^155^	Increased bladder stretch thresholds and bladder pressure; decreased urethral reflexes	
Epithelial cells	*Krt14-Cre*^161^	Reduced frequencies of overall slowly adapting Aβ fibers; decreased sensitivity to gentle touch	Piezo2 is essential to transduce mechanical currents produced by Merkel cells
Gastrointestinal epithelial cells	*Vil-cre*^162^	Decreased mechanosensitive epithelial secretion	
Endothelial cells	*Tie2-Cre*^26^	No effect on oxygen saturation or lung structure	

*AAV* adeno-associated virus, *CNS* central nervous system, *DRG* dorsal root ganglia

Piezo1 is preferentially expressed in endothelial cells as a vital component for mechanotransduction.^63^ The first report of a loss-of-function Piezo1 study demonstrates its indispensable role in the vascular system during embryonic development. Embryos lacking Piezo1 activity resulted in embryotic death at E9.5 as a failure of cardiovascular system development due to impaired endothelial cell alignment.^63^ During lung development, when alveolar pressure increases, pulmonary microvessel endothelial cells stretch to regulate lung vascular permeability and pressure through the mechanosensory channel Piezo1.^132,133^ Piezo1 deletion impaired the ability of endothelial cells to respond to pressure by targeting calcium-dependent cysteine protease calpain^132^ and AJ protein VE-cadherin.^133^ Furthermore, Piezo1 regulates the development and function of lymphatic valves and lymphatic vessels.^134^ Mice lacking lymphatic or endothelial Piezo1 failed to transduce the signals of oscillating shear stress, resulting in retarded body growth, defective lymphatic valve formation, and reduced lymphatic vessel density.^135^ In addition to the circulatory system and respiratory system, Piezo1 participates in angiogenesis^14,62,136^ and blood pressure control.^137,138^ Retailleau et al. showed that Piezo1 enhanced Ca^2+^ influx in endothelial cells to facilitate capillary formation.^14^ Piezo1 deficiency reduced the wall thickness and diameter of arteries in a hypertension model, indicating that the opening of Piezo1 channels affects arterial remodeling.^136^ Furthermore, endothelial Piezo1 senses fluid flow and transmits the signal to adjacent smooth muscle cells, leading to the constriction of smooth muscle and elevation of blood pressure.^137^ Although the detailed mechanisms are still incompletely understood, Piezo1 seems to control calcium flux in endothelial cells in response to an external pressure increase or fluid flow stimulation.

In addition to endothelial cells, Piezo1 was also reported to be highly expressed in other tissues, such as the heart, adipose tissue, pancreas, and bone marrow. Jiang and co-workers^139^ showed that Piezo1 loss in cardiomyocytes impaired Ca^2+^ and reactive oxygen species signaling, resulting in the development of cardiomyopathy in mice. Adipocyte-specific deletion of Piezo1 caused insulin resistance and impaired adipocyte differentiation, leading to hepatic steatosis and inflammation.^140,141^ However, unlike the proinflammatory effect of Piezo1 in adipocytes, genetic deletion of Piezo1 in pancreatic acinar cells inhibited protease activation and autodigestion, leading to the amelioration of cellular necrosis and to acute pancreatic inflammation.^142^ Moreover, mice lacking Piezo1 in myeloid cells were more resistant to pulmonary inflammation.^143^ It would be interesting to further study the systematic influence, such as inflammation, of Piezo1 deletion in a certain tissue type on other tissues.

In addition to the phenotypes from tissue-specific Piezo1 deletion in mice, great efforts have been made to decipher the possible involvement of PIEZO1 in the development of human diseases through cellular models with pharmacological or genetic intervention. PIEZO1 has been shown to participate actively under various neuronal conditions. In a neuroinflammation culture model, lipopolysaccharide induced PIEZO1 upregulation in astrocytes, which inhibited the release of cytokines and chemokines and served as a negative regulator of neuroinflammation.^144^ In another migraine pain model, both Yoda1 and hypo-osmotic solution stimulation could activate neuronal firing, suggesting that PIEZO1 plays a role in peripheral trigeminal nociception, which is related to migraine pain.^145^ In traumatic brain injury (TBI), Piezo1 also contributes to cell mobility through a positive role in blocking Abeta peptides, which are elevated in the central nervous system (CNS) after TBI.^146^ Moreover, in the cultured human cardiomyocyte cell line AC16, PIEZO1 was upregulated under cyclic mechanical simulation.^147^ In prostate cancer cells, shRNA knockdown of PIEZO1 markedly inhibited cell viability, proliferation, and migration and suppressed the growth of prostate tumors in mice.^148^

Compared to Piezo1 in endothelial cells, Piezo2 has been reported to be highly expressed in sensory systems and widely investigated there. Piezo2 is an essential mechanotransduction channel for the sensation of touch, proprioception, tactile allodynia, and mechanical pain in various kinds of neurons.^41,149–151^ Conditional deletion of Piezo2 in CNS neurons decreased the sensitivity to gentle dynamic touch without affecting the sensitivity to noxious pinch.^149^ Dorsal root ganglia (DRG) sensory neurons mediate distinct sensations of touch, proprioception, and mechanical pain.^152^ The deletion of Piezo2 in DRG neurons did not affect the integrity of DRG neurons but impaired gentle touch sensation in mice.^41,94^ Moreover, sensory neuron-specific conditional Piezo2 KO mice exhibited reductions in adapting neurons, mechanosensitive terminals, and nerve terminal impulse discharges in response to mechanical stimulation.^153^

Consistent with its direct roles in neurons, Piezo2 also participates in motor function, bladder control, and other tissues through its neuronal reflections in different subsets of neuronal fibers. Mice lacking Piezo2 in proprioceptive neurons displayed slow growth and poor motor coordination.^22^ Deletion of Piezo2 in the mesencephalic trigeminal nucleus decreased limb coordination.^154^ Loss of Piezo2 in peripheral sensory neurons decreased eyeblinks evoked by harmful mechanical stimuli.^153^ Piezo2 in sensory neurons also participates in urination. The depletion of Aδ- and c-fiber subsets, two primary sensory neuron types, impaired bladder control and the sensation of bladder filling and prolonged the intervals of bladder contractions in mice.^155^ In addition, Piezo2 in various neuronal subsets exhibits diverse functions in controlling respiration. The deletion of Piezo2 in jugular, trigeminal, and DRG caused respiratory distress in mouse pups, which could not survive 24 h after birth.^26^ Mice lacking Piezo2 in vagal and spinal sensory neurons survived but lived with decreased vagal nerve firing and abnormally increased lung tidal volume.^26^ However, the conditional deletion of Piezo2 in nodose ganglia or endothelial cells did not affect the lung structure or oxygen saturation in mice.^26^

**Fig. 5:**
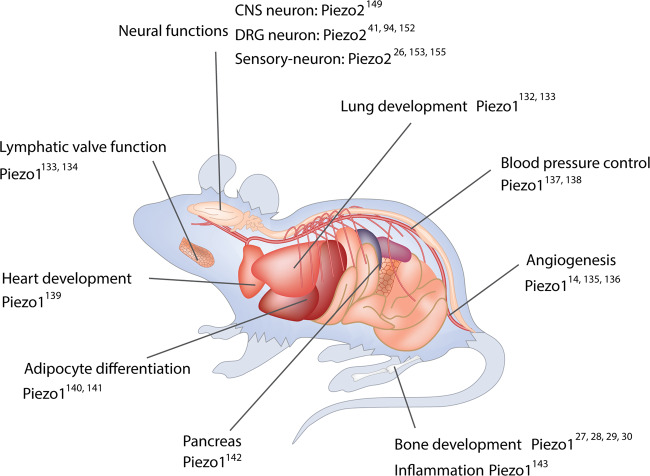
Piezo studies in mice. Piezo1 is widely expressed in multiple tissues with a preference for the endothelium. Studies focused on tissue-specific deletion of Piezo1 in experimental mice demonstrate the importance of Piezo1 in regulating lung development, angiogenesis, blood pressure control, bone development, lymphatic valve function, heart development and adipocyte differentiation and pancreas functions. Piezo2 is highly expressed in neurons. Current studies reveal the great contribution of Piezo2 to regulating mechanotransduction in central nervous system (CNS) neurons, dorsal root ganglia (DRG) neurons, and other sensory neurons

In addition to the high expression of Piezo1 in endothelial cells and Piezo2 in neurons, Piezo1 and Piezo2 displayed some functional redundancy in certain tissues. Zeng et al.^156^ found that Piezo1 and Piezo2 double KO in epibranchial placode-derived ganglia diminished baroreflex and nerve activity, resulting in hypertension, while Piezo1 or Piezo2 single-KO mice did not show these phenotypes. Piezo2 is predominantly expressed in sensory tissues, including DRG neurons.^149,157,158^ Fernandez et al.^153,159^ reported that the deletion of either Piezo1 or Piezo2 in mammalian DRG neurons inhibited the inward calcium current and mechanosensation, which was regulated by the activation of TRPV1. Furthermore, Piezo proteins receive and transduce mechanical forces in epithelial tissues. Piezo1 mediates touch and pressure sensitivity in pancreatic acinar cells, one of the main epithelial cells in the pancreas.^142^ Knockdown of Piezo1 in bladder urothelial cells reduced the sensitivity and signal transduction of mechanical forces in the bladder.^160^ Moreover, epithelial Piezo2 KO led to disorders in sensory functions of gentle touch^155,161^ and in secretion functions.^162^ Taken together, these studies demonstrate the indispensable roles of Piezo proteins in the development and function of various tissues (Fig. 5).

It is worth mentioning that Piezo2, through its expression in proprioceptive neurons, regulates skeletal integrity.^131^ While blocking Piezo2 expression through *Prx1-Cre*, *Col1a1-Cre* or *Col2a1-Cre* did not cause abnormal spinal alignment in mice,^131^ deletion of Piezo2 in proprioceptive neurons using *PValb-Cre* resulted in dramatic spine malalignment and misshapen joints, which was due to the development of abnormal skeletal muscle functions through an impaired proprioceptive system.^131^ These phenotypes highly resemble those observed in human diseases linked to *PIEZO2* mutations^131^ and highlight the importance of the neuronal regulation of skeletal health. Another study published in a current preprint by Chen et al. showed that Piezo1 expression in the endothelium was essential for effective bone fracture healing.^163^ Endothelial cell-specific Piezo1 deletion with *Cdh5-Cre* (vascular endothelial cadherin or cadherin 5) resulted in impaired bone fracture repair.^163^ By utilizing the Piezo1-tdT reporter mouse line, Chen and co-workers showed that Piezo1 was colocalized to Endomucin, a membrane-bound endothelial cell glycoprotein, in endothelial cells from the bone vasculature.^163^ Conditional inactivation of Piezo1 in endothelial cells by tamoxifen-inducible deletion (*Piezo1*^*Cdh5-ER2*^) had no effects on bone mass or bone structure in mice but significantly delayed fracture union with incomplete bridging in a stabilized femur fracture model.^163^ This healing defect could result from impaired new blood vessel formation through the PI3K-AKT pathway in KO mice.^163^ These results suggest that Piezo proteins function in diverse tissues.

## Piezo proteins in health and diseases

Increasing evidence from animal models highlights the diversity and importance of Piezo proteins in organ development and homeostasis (Fig. 5). It should be noted that some functions of Piezo1 and Piezo2 have been assessed only in mouse tissues and even seem to be contradictory in different species. For example, Koser et al.^18^ reported that knockdown of Piezo1 using morpholino in *Xenopus* led to slowed axon growth and pathfinding abnormalities. However, Song et al.^164^ reported that DmPiezo KO in *Drosophila* and sensory neuron-specific KO of Piezo1 in mice exhibited no obvious defects in axon guidance or patterning during development but displayed accelerated axon regeneration after injury. These contradictory results may be attributed to species differences and/or compensatory effects. However, it indeed reveals the important role of Piezo1 in axon growth and suggests that this function should be further assessed in humans, who may benefit from axon regeneration and nerve repair.

**Fig. 6:**
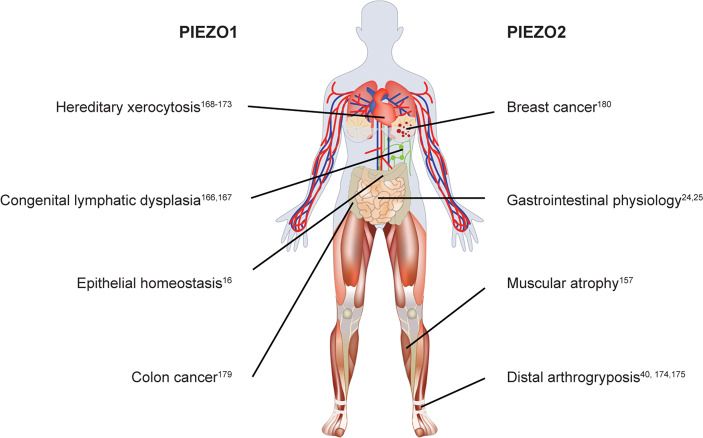
Piezo proteins in health and diseases in humans. In healthy humans, Piezo proteins play an important role in epithelial homeostasis and gastrointestinal physiology. Under pathological conditions, loss-of-function mutations in the human *PIEZO1* gene are linked to autosomal-recessive congenital generalized lymphatic dysplasia of Fotiou (GLDF). Gain-of-function mutations in the human *PIEZO1* gene cause hereditary xerocytosis (HX). Loss-of-function mutations in the human *PIEZO2* gene result in an autosomal-recessive syndrome of muscular atrophy. Gain-of-function mutations in the human *PIEZO2* gene lead to autosomal-dominant distal arthrogryposis (DA). In addition to hereditary human diseases, abnormal PIEZO protein expression is associated with colon cancer and breast cancer

Emerging evidence shows that PIEZO proteins play important roles in human health and diseases (Fig. 6).^10,47^ Recently, whole-exome sequencing of patients with varied disorders across different ethnic and geographical backgrounds identified several loss- and gain-of-function mutations in *PIEZO1* or *PIEZO2* genes.^11,12,165^ For example, loss-of-function mutations in the human *PIEZO1* gene are linked to autosomal-recessive congenital generalized lymphatic dysplasia of Fotiou, which is characterized by widespread lymphoedema affecting all parts of the body.^166,167^ Gain-of-function mutations in the human *PIEZO1* gene cause hereditary xerocytosis, also known as dehydrated stomatocytosis, characterized by primary erythrocyte dehydration and compensated hemolytic anemia.^168–173^ Loss-of-function mutations in the human *PIEZO2* gene result in an autosomal-recessive syndrome of muscular atrophy, often accompanied by arthrogryposis, perinatal respiratory distress, and scoliosis.^157^ Gain-of-function mutations in the human *PIEZO2* gene lead to three clinical types of autosomal-dominant distal arthrogryposis (DA), including DA3 [also known as Gordon syndrome (GS)], DA5, and Mardene–Walker syndrome (MWS).^40,174,175^ GS is characterized by multiple contractures of the limbs and cleft palate, and DA5 is related to additional ocular manifestations. MWS shares a musculoskeletal phenotype similar to DA5 and GS but is further characterized by hindbrain malformations and developmental delay.^176^ In addition to hereditary human diseases, PIEZO proteins are tightly associated with several cancers.^177,178^ Sun et al.^179^ reported that PIEZO1 was upregulated in colon cancer tissues, which was closely correlated with poor prognosis of colon cancer, and that PIEZO1 overexpression in vitro promoted the migration and metastasis of colon cancer cells. Lou et al.^180^ found that PIEZO2 was downregulated in breast cancer tissues and could be used as a prognostic biomarker of breast cancer.

In addition to mutation-related diseases, clinical studies have revealed abnormal Piezo protein expression in several pathological conditions. Jiang and colleagues^139^ showed that heart samples from patients with hypertrophic obstructive cardiomyopathy expressed a significantly higher level of *PIEZO1* mRNA than those from normal human hearts. This result indicated that autonomic upregulation of PIEZO1 in cardiomyocytes contributed to cardiomyopathy through altered mechanical stress conditions in these patients. Moreover, the expression of PIEZO1 proteins varies in human bone during osteopenia and aging. Sun et al.^28^ reported that the mRNA and protein levels of PIEZO1 were significantly lower in osteoporosis patients than in normal patients. Zhou et al.^29^ found that the gene expression of PIEZO1 and PIEZO2 from human bone MSCs was negatively correlated with age. Together, these results demonstrate the importance of *PIEZO* genes in inherited diseases and PIEZO proteins in different pathological conditions. Considering the wide involvement and species-specific functions of PIEZO proteins in living organisms, large-scale studies are needed to reveal the functions of PIEZO1 and PIEZO2 in physiological and pathological processes. The underlying molecular mechanisms can enhance our understanding of PIEZO proteins in human health and contribute to the discovery of effective therapies for related diseases.

## Conclusions and perspectives

With 10 years of great efforts from scientists worldwide, the mystery of Piezo proteins has gradually been unraveled. The essential functions and unique structures of Piezo proteins have been reported in various tissue backgrounds from different species. There are several questions that deserve further detailed investigation. First, the molecular mechanisms underlying Piezo1-mediated mechanotransduction in bone remain elusive and require further investigation. Second, whether and how the expression of Piezo1 in skeletal cells or the expression of Piezo2 in neurons contributes to bone fracture healing remains to be determined. Third, there are several puzzling but intriguing questions that link Piezo proteins to OA pathogenesis and OA pain. For example, what are the functions of Piezo1 channels in chondrocytes under physiological and pathological loading conditions? Is Pieoz2 expression in sensory neurons associated with OA pain, and if so, how? Together with these questions, it is encouraged to involve more pharmacological animal and clinical studies to develop new drugs or novel therapeutics for relevant pathologies.
